# Cardiopulmonary and skeletal muscle strategies underlying exhaustive exercise in adults with glycogen storage disease type III

**DOI:** 10.14814/phy2.70771

**Published:** 2026-02-22

**Authors:** F. Lanfranconi, L. Peli, L. Pollastri, A. Ferri, L. Tremolizzo, E. Conti, F. Pieruzzi, G. Miserocchi, E. Beretta, M. Marzorati, W. Zardo, S. Gasperini, R. Pretese, S. Paci, C. Capelli, R. Mariani, A. Cattoni, A. C. Balduzzi, R. Parini

**Affiliations:** ^1^ Centro Maria Letizia Verga, Fondazione Monza e Brianza per il bambino e la sua mamma Monza Italy; ^2^ AMSD Lecco, Federazione Medico Sportiva Italiana Rome Italy; ^3^ Institute for Health and Sport (IHES) Victoria University Melbourne Victoria Australia; ^4^ Neurology Department Fondazione IRCCS San Gerardo dei Tintori Monza Italy; ^5^ School of Medicine and Surgery University of Milano‐Bicocca Monza Italy; ^6^ Nephrology Department Fondazione IRCCS San Gerardo dei Tintori Monza Italy; ^7^ Istituto Tecnologie Biomediche, Consiglio Nazionale delle Ricerche, LITA Segrate Italy; ^8^ Pediatric Oncology Unit Fondazione IRCCS Istituto Nazionale Tumori Milan Italy; ^9^ Pediatrics Department Fondazione IRCCS San Gerardo dei Tintori Monza Italy; ^10^ ASST Santi Paolo e Carlo‐Presidio San Paolo Milan Italy; ^11^ Department of Pathophysiology and Transplantation University of Milano Milan Italy; ^12^ Internal Medicine Department, Rare Diseases Unit Fondazione IRCCS San Gerardo dei Tintori Monza Italy; ^13^ San Raffaele Telethon Institute for Gene Therapy (SR‐Tiget) Milan Italy

**Keywords:** exercise, glycogen storage disease type III, near infrared spectroscopy, oxidative metabolism, skeletal muscle

## Abstract

People with glycogen storage disease type III (GSDIII‐p) have a remarkably reduced exercise tolerance. Aim of this study was to analyze the oxygen transport‐utilization chain strategies adopted by GSDIII‐p during exercise. Nine GSDIII‐p (39.4 ± 10.0 year, 33% female) and 11 healthy controls (CTRL), age and gender matched, underwent an incremental cardiopulmonary exhaustion test (CPET) to assess peak heart rate (HR), blood lactate [La]p and vastus lateralis O_2_ fractional extraction (ΔHHb/isch) using near‐infrared spectroscopy. Patterns of breathing (PBr) were assessed accordingly by analyzing pulmonary O_2_ uptake (V̇O_2_), tidal volume (Vt), respiratory frequency (Rf), end‐tidal CO_2_ (PETCO_2_) and alveolar ventilation (V̇A). GSDIII‐p exhibited significantly (*p* < 0.05) lower peak values of V̇O_2_, pulmonary ventilation (V̇E) [La] and ΔHHb/isch compared to CTRL (1.7 ± 0.7 vs. 3.2 ± 1.1 L/min, 50.5 ± 19.8 vs. 113.6 ± 40.4 L/min, 1.8 ± 0.7 vs. 7.6 ± 3.0 mmol/L and 39.1% ± 9.9% vs. 74.8% ± 36.6%, respectively). The range of peak V̇O_2_ values for GSDIII‐p, compared to the predicted values for age and sex, was between 79% and 35%. Both GSDIII‐p and CTRL were arbitrarily divided into 4 groups according to individual V̇E values. GSDIII‐p with exercise intolerance relied on increased Rf with inadequate Vt adaptation to maintain V̇E and reduce PETCO_2_, with low V̇A values and low to moderate workloads tolerance. Reduced exercise tolerance in GSDIII‐p is related to respiratory and skeletal muscle inefficiencies. GSDIII‐p strong heterogeneity evaluated throught CPET provides insights into clinical management.

## 1 INTRODUCTION

Glycogen storage disease type III (GSD‐III; OMIM #232400) also known as “limit dextrinosis,” “Cori” or “Forbes” disease, is a rare autosomal recessive glycogen storage disorder caused by mutations in the amylo‐1,6‐glucosidase gene (AGL) that result in a deficiency of the glycogen debranching enzyme (GDE) (Dagli et al., 2010). To date, over 180 different AGL gene (MIM #610860) pathogenic variants have been reported worldwide, reflecting a high degree of genetic heterogeneity (Dagli et al., 2010). Due to pathogenic variations in the AGL gene, GDE deficiency or reduced activity leads to various degrees of accumulation of limit dextrin‐like molecules in the cytoplasm of hepatocytes, myocytes, and other tissues.

The global prevalence is estimated at approximately 1 in 100,000, with a higher occurrence observed among specific, geographically isolated populations (Kishnani et al., 2010). The first 4 Italian people with GSD‐III (GSDIII‐p) were reported and confirmed by molecular analysis in 1999 (Hadjigeorgiou et al., 1999). The Italian Glycogenosis Association reports that the prevalence of the condition in Italy is comparable to the global prevalence (https://www.aiglico.it/tipo‐3/, accessed on September, 28, 2025) and this indicates that the number of new diagnoses each year will likely remain at approximately 3–4.

Clinically, GSDIII‐p are affected in infancy or early childhood with hepatomegaly, ketotic hypoglycemia, hyperlipidemia, and growth delay. In glycogen storage disease type IIIa (GSDIIIa), GDE activity is deficient in both skeletal muscle and liver (Preisler, Laforêt, et al., 2015), accounting for 85% of GSDIII‐p. The remaining 15% have liver disease alone without muscle involvement and are referred to as GSDIIIb. Muscle weakness, a manifestation of varying degrees of myopathy in individuals with GSDIIIa, is typically minimal in childhood. However, it can become more severe in adulthood, with weakness and atrophy of limb muscles typically developing in the 3rd to 4th decade of life. In adulthood, GSDIII‐p can develop severe cardiomyopathy as well as liver fibrosis/cirrhosis and quite often hepatocellular carcinoma (Lucchiari et al., 2007; Rossi et al., 2020; Shen et al., 1996). Weakened respiratory muscles, due to the accumulation of abnormal glycogen in muscle tissue, can lead to difficulties in breathing, impaired secretion clearance, and an increased risk of infections like pneumonia (Lee et al., 2011). The extent to which respiratory inefficiency can contribute to exercise intolerance in GSDIII‐p is still unknown. Inherited metabolic diseases may affect the upper and lower airways, the thoracic wall and all muscles involved in pulmonary ventilation (Lee et al., 2011). Alveolar hypoventilation due to respiratory muscle dysfunction and/or abnormal respiratory mechanics related to enlarged abdominal organs may develop in glycogenosis, though mainly in GSD type I and II, but only few case reports inquire about GSDIII‐p (Lee et al., 2011). In addition, the likely inefficiency of the respiratory muscular oxidative pathway may substantially contribute to exercise intolerance.

GSDIII‐p had clinical and biochemical heterogeneity among different individuals in using the glycogenolytic chain, reflecting genotype–phenotype heterogeneity, affecting their exercise tolerance (Preisler et al., 2013). Symptoms of pain and excessive fatigue experienced by GSDIII‐p can most likely be attributed to an energy deficit in the muscles involved in exercise (Preisler et al., 2013). In another study, Preisler, Laforêt, et al. (2015) also provided evidence that GSDIII‐p have a severely limited ability to break down glycogen during exercise, resulting in an energy deficit and a compensatory increase in fat oxidation. While blocked glycogenolysis caused inadequate substrate supply to the mitochondria, combined measurements suggested that altered perfusion was also responsible for impaired post‐exercise phosphocreatine recovery and could contribute to exercise intolerance in GSDIII Wary et al. (2010).

The therapeutic options aim to improve the quality of life by alleviating signs and symptoms and many authors argue that improving individual exercise tolerance is one of the primary outcomes that should be addressed early after diagnosis and in the follow‐up process (Preisler, Laforêt, et al., 2015). Still, a consensus has not been reached regarding the optimal type and intensity of exercise for this population (Bordoli et al., 2022). Home‐based, remotely supervised programs combining moderate‐intensity endurance and resistance exercises have been recently introduced as a promising solution to face exercise intolerance in GSDIII‐p (Bustos‐Sellers et al., 2025).

Investigating oxidative metabolism in GSDIII‐p could be essential to quantify functional limitations that can significantly affect individual exercise tolerance and performance and set up exercise programs for managing muscle weakness and exercise intolerance issues. During an ergometric evaluation, or cardiopulmonary exercise test (CPET), peak O_2_ consumption (V̇O_2_peak), a descriptor of the overall efficiency of the O_2_ transport and utilization chain, that is, the individual exercise tolerance, can be assessed. Impairment of this process, at either the central or peripheral level, can result in adaptive strategies to maintain O_2_ for the ultimate goal of supporting ATP production through the oxidative metabolism pathway. Preisler et al. (2013), Hennis et al. (2022) and Bustos‐Sellers et al. (2025) found V̇O_2_peak is lower in GSDIII‐p than predicted values, based on their demographic data, but the need of investigating peripheral muscular oxygenation during exercise is warranted if we aim to describe the peripheral impairment of oxidative metabolism. Near‐infrared spectroscopy (NIRS) during CPET is a noninvasive technique that measures skeletal muscle fractional O_2_ extraction and can gain pathophysiological and diagnostic insights as well as evaluate the effects of therapeutic interventions (Grassi & Quaresima, 2016).

Therefore, the aims of this study in GSDIII‐p were: (1) to evaluate the O_2_ transport‐utilization chain during exhaustive exercise using non‐invasive methods; (2) to assess the central (cardiopulmonary) and peripheral (skeletal muscle) determinants of exercise tolerance; (3) to determine whether different patterns of breathing provide different strategies to support exercise tolerance; and (4) to relate clinical evaluation scores with physiological outcomes measured during CPET.

## 2 METHODS

### 2.1 Volunteers

GSDIII‐p were recruited through the Italian Glycogenosis Association and evaluated in an Italian center for rare genetic disorders. The experimental protocol was reviewed and approved by the institutional ethical committee (research project GSDIIIDIET, University of Milano Bicocca, ethical approval #253) and was conducted in accordance with the Declaration of Helsinki. All GSDIII‐p were informed about the aims and details of the study and signed a written informed consent form. All the GSDIII‐p had biochemical diagnosis with enzyme testing on erythrocytes and 8 had molecular testing with identification of two pathogenic variants each (Table 1). Eleven healthy participants, sex‐ and age‐matched, who participated in a previous research project (SOSPESI, University of Milan Bicocca, ethical approval #4566), were included as a control group (CTRL). The CTRL group consisted of both fit and sedentary individuals to identify the most effective and average strategies for managing an exhaustion exercise from the perspective of respiratory system efficiency.

**TABLE 1:** Demographic and clinical characteristics of people with GSD‐III and CTRL.

	GSDIII‐p	CTRL
Age, years	39.4 ± 10.0 (27–54)	41.6 ± 12.2 (26–60)
Weight, kg	80.9 ± 14.7 (66–105)	69.6 ± 14.6 (50–97)*
Height, cm	176.9 ± 8.6 (165–192)	175 ± 11.1 (160–193)
Body mass index, kg/m^2^	25.7 ± 3.15 (22–28)	22.9 ± 2.4 (19–27)*
Fat mass, %	16.0 ± 4.8 (9–22)	11.7 ± 4.6 (5–23)*
Free fatty mass, kg	67.0 ± 13.8 (53–91)	62.8 ± 13.2 (43–80)*
Sex, female/male	3/6	4/7
Diagnosis, definite/probable	9/0	/
Onset, age, years	5.2 + 6.7 (0.5–10)	/
AGL gene variants, num	Nonsense, 7	/
	Frameshift, 3	/
	Missense, 3	/
	Insertion, 1	/
	Site splicing, 1	
	Deletion, 1	/
Non invasive ventilation, no/yes	9/0	/
Diet, mildly hyperproteic/no	8/1	/
Blood urinary ketons, no/yes	9/0	/

*Note*: Values are expressed as mean and standard deviation (ranges).

Abbreviation: GSDIII‐p = people with GSD‐III.

* *p* < 0.05.

### 2.2 Clinical evaluation

All GSDIII‐p underwent a medical evaluation the day before the exercise testing to exclude the presence of acute cardiopulmonary and/or infectious diseases. Specifically, their medical examination included: (1) occupational, exercise, and sport history; (2) dietary intake was assessed through the collection of food diaries, followed by a dietitian‐led interview to verify entries and improve accuracy, allowing estimation of habitual total energy intake and macro‐ and micronutrient consumption; (3) after an overnight fast, venous blood samples were collected and analyzed using routine standardized methods in the accredited main regional hospital laboratory. Measurements included fasting glucose, insulin, lipid profile (total, LDL, and HDL cholesterol, triglycerides), creatine kinase (CPK), uric acid, liver and kidney function parameters, complete blood count, NT‐proBNP, and myoglobinaemia. Urinary ketone bodies were assessed using urine ketone dipstick; (4) serum concentrations of muscle stress–related cytokines were quantified using commercially available enzyme‐linked immunosorbent assay (ELISA) kits, following the manufacturers' protocols (Quantikine Colorimetric Sandwich ELISA kits, R&D systems, Minneapolis, USA). Interleukin‐6 (IL‐6) (Cat. No. HS600B, sensitivity: MDD 0.016–0.110 pg/mL, mean MDD 0.039 pg/mL), tumor necrosis factor‐α (TNF‐α) (Cat. No. HSTA00E, sensitivity: MDD 0.011–0.049 pg/mL, mean MDD 0.022 pg/mL), brain‐derived neurotrophic factor (BDNF) (Cat. No. DBD00, sensitivity: MDD less than 20 pg/mL), and myostatin (GDF‐8/myostatin) (Cat. No. DGDF80, sensitivity: MDD 0.922–5.32 pg/mL, mean MDD 2.25 pg/mL). All assays were performed according to the manufacturers' instructions, including appropriate standards, controls, and quality checks; (5) complete cardiologic evaluation including clinical cardiologic examination, electrocardiogram and echocardiography.

An anthropometric evaluation was performed to measure body mass index (BMI). The 7‐site skinfold plicometry and the Siri formula were used to calculate body composition (fat mass‐FM%, fat‐free mass‐FFM) (Jackson & Pollock, 1978; Jackson et al., 1980).

All GSDIII‐p performed the Quick Motor Function test (QMFT) to assess disease severity (van Capelle et al., 2012). The QMFT is a brief, 16‐item assessment tool designed to evaluate and monitor motor function and clinical severity in people with conditions like Pompe disease and Duchenne muscular dystrophy. QMFT explores a range of motor abilities, including the ability to stand, squat, and raise arms. The total QMFT score ranges from 0 to 64 (16 items × 4 points each), with higher scores indicating greater motor function.

Each GSDIII‐p underwent a spirometry test in both orthostatism and clinostatism to determine forced vital capacity (FVC) and forced expiratory volume in 1 s (FEV1). A visual assessment of the expected spirometry curves was made, and the average of 3 consecutive and repeatable measurements was used. All these variables were expressed in terms of predicted values based on race (Caucasian), age, sex, and height.

### 2.3 Conventional echocardiography

An echocardiogram was obtained in the standard precordial positions using digital echocardiography equipment (Aloka ProSound SSD Alpha 10, Tokyo, Japan) with 1‐5 MHz transducers. We followed the recommendations for standard measurements from M‐mode echocardiograms (Lang et al., 2005). Instantaneous measurements were made over three cardiac cycles and the average values of the following were obtained from each participant: interventricular septum diastolic thickness (IVSd), left ventricular posterior wall thickness at end‐diastole (LVPWd), and left ventricular end diastolic dimension (LVEd). Left ventricular mass (LVM) was calculated according to the formula modified by Devereux et al. (1986) using the American Society of Echocardiography convention (de Simone et al., 1995) and standardized to a power of height (2.7) to account for allometric scaling in cardiovascular studies (LVMI).

### 2.4 Exercise tolerance evaluation

Each participant underwent a CPET to exhaustion on a cycle ergometer (ErgomedicMonark LC6: Monark, Varberg‐Sweden) under medical supervision. Electrocardiography was used to determine heart rhythm and rate (HR) by 12‐lead monitoring (Quark C12x: Cosmed, Roma‐Italy) and arterial oxygen saturation (SaO_2_) was continuously recorded by finger pulse oximetry (RAD 9 Signal Extraction Pulse Oximeter: Masino Corporation, Irvine, California, USA). Participants were carefully assessed to be in a resting state for 2–6 min prior to testing by measuring HR, tidal volume (Vt) and respiratory frequency (Rf). Breath‐by‐breath pulmonary ventilation in BTPS (V̇E), V̇O_2_, CO_2_ output (V̇CO_2_), and the partial pressure of CO_2_ at the end of an exhaled breath (PETCO_2_) were measured using a metabolic cart (Vmax Spectra 229: Sensormedics, Yorba Linda, California, USA). After 2 min of unloaded pedaling, the ramp slopes (3–5–10–15 W min^−1^) were empirically selected according to the individual's habitual activities, as determined by a pretest interview, and the power increased until voluntary exhaustion was reached. V̇O_2_peak was considered at “exhaustion” when the participant was unable to maintain the pedaling frequency despite vigorous encouragement from the operators. The averaged last 30 s of exhaustion values were considered as peak of exercise. The Borg scale (from 6 to 20 score) was used to assess subjective perceived exercise effort during CPET and at exhaustion. At rest and at various times (1, 3, and 5 min) during recovery, 20 μL of capillary blood was obtained from a preheated earlobe for the determination of blood lactate concentration by an enzymatic method (Biosen C‐line Clinic: EKF Diagnostic, Cardiff‐England). The highest value measured during recovery was taken as the lactate peak concentration ([La]p).

On the basis of the V̇Epeak value achieved, participants were arbitrarily divided in four groups: group 1 (1st quartile: >100 L/min), group 2 (2nd quartile: from 70 to 99 L/min), group 3 (3rd quartile: from 41 to 69 L/min), and group 4 (4th quartile: < 40 L/min).

### 2.5 Near infrared spectroscopy

NIRS is a non‐invasive method that allows the monitoring of muscle oxygenation on the principle that the near‐infrared (NIR) light absorption characteristics of hemoglobin (Hb) and myoglobin (Mb) depend on their O_2_ saturation. The absorption characteristics of light at 780 and 850 nm depend on the relative oxygenation of Hb and Mb. Details on the method can be found in previous studies from our group (Grassi et al., 2007). In the present study a NIR continuous‐wave photometer (Nimo: Nirox, Brescia, Italy) was utilized. Data were expressed as a variation of absolute units, Δ[O_2_Hb] and Δ[HHb], from the baseline value recorded after a 2‐min light warm up and as a function of time. Therefore, decreases in Δ[O_2_Hb] and corresponding increases in Δ[HHb] were interpreted as evidence of relative muscle deoxygenation and, conversely, as evidence of improved oxygenation. Since Δ[HHb] is closely associated with changes in venous O_2_ content, it is believed to be a sensitive measure of relative tissue deoxygenation due to O_2_ extraction (Grassi & Quaresima, 2016).

The NIRS probe was firmly placed on the skin over the lower third of the right vastus lateralis muscle (∼10 cm above the proximal border of patella and 3 cm lateral to the midline of the thigh), and secured with a small belt of Velcro straps. Elastic bandages were put around the muscle probe to prevent contamination from localized light. Pen‐marks were made on the skin to indicate the margins of the plastic spacer, allowing for a check on any downward sliding of the probe during exercise. Once secured in place, no sliding of the probe was detected in any of the participants. According to the Monte Carlo method of simulation, based on skin and muscle scattering and absorption characteristics for NIR light, in in‐vivo measurements, a source‐detector spacing of 20 mm is enough for the NIR light to pass through the muscle layer, even when the adipose tissue thickness is 15 mm. All participants underwent plicometry a few min before the NIRS probe was placed, and all had an adipose tissue thickness of <15 mm. Data were recorded at 2 Hz. Muscle Δ[HHb] data were expressed as a percentage of the maximal deoxygenation reference point obtained by a post exercise leg‐cuff ischemia procedure (Δ[HHb]/Δ[HHb]isch). Arterial ischemia was induced by inflating a cuff to 200–300 mmHg (according to the participant's body mass index) for 2–3 min while they were sitting on the medical bed and it was confirmed when a simultaneous increase in [HHb] and a decrease in [O_2_Hb] reached a plateau.

### 2.6 Estimating inspiratory elastic work

A simple approach to estimating inspiratory elastic work was proposed, and it proved helpful for comparing energy expenditure when changing the breathing pattern during exercise in people with metabolic syndrome (Passoni et al., 2015). Considering the linearity of the overall volume‐pressure relationship of the respiratory system between the functional respiratory capacity up to end‐inspiration, the elastic work (InEW) done by the inspiratory skeletal muscles can be defined as the product of the change in lung volume ($V_{t}$) and the corresponding change in distending pressure (ΔP), namely:

$$\text{In}\mathrm{EW}=V_{t}\cdot \mathrm{\Delta P}$$

Defining total respiratory compliance as follows:

$$\mathrm{Crs}=V_{T}/\mathrm{\Delta P}$$

one has

$$\text{In}\mathrm{EW}=\frac{1}{2\mathrm{Crs}}{V_{t}}^{2}$$

and knowing respiratory frequency (Rf), the elastic work per minute (having the dimension of power) becomes:

$$\text{In}\mathrm{EW}=\frac{1}{2\mathrm{Crs}}{V_{t}}^{2}\cdot \mathrm{Rf}$$

Assuming factor Crs unchanged for each participant at rest and during exercise (Passoni et al., 2015) the product $\frac{1}{2}{V_{t}}^{2}\cdot \mathrm{Rf}$ can provide an index of the relative changes in InEW, on comparing different patterns of breathing.

### 2.7 Estimating alveolar ventilation

PETCO_2_ can be used to estimate V̇A, or the amount of air that reaches the alveoli for gas exchange. This estimation is based on the principle that PETCO_2_ reflects alveolar CO_2_, and V̇A is directly related to CO_2_ elimination. The following formula was used to calculate V̇A (Miserocchi, 2009):

$${\text{PETCO}}_{2}=\frac{\dot{V}{\mathrm{CO}}_{2}}{\dot{V}A}\cdot \left(\text{Pbar}-47\right)$$

and if we solve for V̇A

$$\dot{V}A=\frac{\dot{V}{\mathrm{CO}}_{2}}{{\text{PETCO}}_{2}\;}\cdot \left(\text{Pbar}-47\right)$$

### 2.8 Statistics

Values were expressed as mean ± (SD). The sample size was limited by the maximum number of GSDIII‐p who met the inclusion criteria in Italy. D'Agostino and Pearson omnibus normality test was used to check if values came from a Gaussian distribution. The statistical significance of the mean values difference between groups was evaluated by unpaired two‐tailed Student's *t*‐test where the normality test was passed; otherwise, the Mann–Whitney test was used (95% confidence level). Regression and correlation analyses were performed using the least squared residuals method. The level of significance was set at *p* < 0.05. The fitting of nonlinear equations was performed by the Levenberg–Marquardt method. All statistical analyses were performed using a commercially available software package (Prism 8.0: GraphPad, La Jolla, CA, USA).

## 3 RESULTS

### 3.1 Characteristics of GSDIII‐p and of volunteers

The demographic, anthropometric and genetic data of GSDIII‐p are shown in Table 1, as well as those of the CTRL. There was a statistically significant difference in BMI and fat mass, with GSDIII‐p having higher values compared to CTRL. One female with GSD‐III (age at enrollment 44 years) underwent liver transplantation for cirrhosis at the age of 30 years. One GSDIII‐p was using a walking aid. The abdominal examination showed no or very mild hepatomegaly in all participants, with the liver edge at 0–2 cm from the costal margin. All the GSDIII‐p had a normal caloric intake; they all were treated with uncooked cornstarch during childhood and 8 out of 9 followed a mildly hyperproteic diet according to Italian recommended dietary allowance (protein >1 g/kg, CHO 45%–60%, lipids 20%–35%). The only participant who had a normoproteic diet (protein 0.9 g/kg) was the female with transplanted liver. All GSDIII‐p were sedentary or involved in recreational activities.

GSDIII‐p and CTRL were divided in the 4 arbitrarily groups (see Section 2), while exhibiting different patterns of breathing. Their individual blood exams, echocardiographic and spirometric values are presented in Table 2. GSDIII‐p and CTRL allocated to the 4 patterns of breathing with their strategies to tolerate exhaustive exercise were defined as follows (see also Table 3): very good responders, good responders, poor responders and exercise intolerants. All but 1 of the 9 GSDIII‐p had elevated levels of transaminases (from 2 to 6 times normal levels) and CPK (from 10 to 50 times normal levels); myoglobinemia was elevated in all but 1 participant (from 2.5 to 19 times). In addition, pseudocholinesterase levels were within the normal range in all GSDIII‐p and N‐terminal pro‐B‐type natriuretic peptide (NT‐proBNP) was elevated in 3 GSDIII‐p (from 1.5 to 30 times). Echocardiogram evaluation showed that all but 1 GSDIII‐p had a mild increase in interventricular septum thickness. According to reference limits and partition values of left ventricular mass and geometry, left ventricular mass (g/m^2^) was abnormal in 5 participants: severely abnormal in 2 GSDIII‐p (1 female, 1 male); moderately abnormal in 2 (1 female, 1 male), and mildly abnormal in 1 female. Normal ejection fraction was maintained within the limits in all GSDIII‐p.

**TABLE 2:** Individual clinical data (resting condition) of people with GSD‐III in the four study groups divided according to V̇E values at peak.

	Blood test			Echocardiogram			Spirometry							
	CPK	GOT/GPT	Myostatine	Septal thickness	LVM	EF	FVC	FVC	FEV1	FEV1	FVC	FVC	FEV1	FEV1
							Standing (upright) position				Laying down (supine) position			
	(U/L)	(U/L)	ng/mL	mm	g/m^2^	%	L	%	L	%	L	%	L	%
Very good responders														
GSDIII9	98	12/25	/	0.9	153	68	6.5	119	5.4	119	5.8	105	4.7	104
Good responders														
GSDIII8	5913	161/153	1.7	1.5	307	69	6.1	102	5.2	104	6.3	104	5.1	102
Poor responders														
GSDIII7	1399	104/80	1.7	2.5	369	73	3.0	90	2.5	86	2.9	87	2.4	84
GSDIII6	2675	169/105	2.2	1.1	194	66	5.9	108	4.8	110	5.7	105	4.6	106
GSDIII5	1075	82/45	1.8	1.3	223	67	5.3	119	4.1	116	4.8	108	3.9	109
Exercise intolerants														
GSDIII4	2737	121/104	2.1	1.3	169	63	5.8	106	4.2	99	5.8	106	3.8	89
GSDIII3	810	48/51	1.5	1.2	167	70	3.7	103	2.9	95	3.7	103	2.8	91
GSDIII2	1454	75/51	2.1	1.2	201	67	2.9	88	2.2	80	2.6	81	2.0	72
GSDIII1	9032	196/92	4.7	1.1	163	71	5.5	114	4.5	115	5.3	111	4.2	107

Abbreviations: EF, ejection fraction; FEV1, forced expiratory volume in the first second; FVC, forced vital capacity; LVM, left ventricular mass.

**TABLE 3:** Ventilation data at peak of exercise in the four study groups stratified according by V̇E values at peak.

VE peak	Vt peak	Resp. work peak	Model	Vt at X0	GSDIII‐p	CTRL
L/min	L	W		L	#	#
Very good responders						
>100	>2.4	>120	Non‐linear regres.	2.4	1	7
Good responders						
70<X<99	1.9<X<2.3	80<X<119	Non‐linear regres.	1.5	1	2
Poor responders						
41<X<69	1.3<X<1.8	16<X<79	Linear regres.	/	3	2
Exercise intolerants						
<40	<1.2	<15	Linear regres.	/	4	0

*Note*: Each individual with GSD‐III fell in one of the 4 patterns of breathing.

Abbreviations: V̇E, pulmonary ventilation; Vt at X0, threshold of the relationship between V̇E vs. Vt; Vt, tidal volume.

The averaged serum levels of myokines were: myostatine 3.0 ± 2.0 ng/mL, IL‐6 4.7 ± 4.5 pg/mL, TNF‐α 1.9 ± 3.1 pg/mL, BDNF 51.9 ± 3.0 ng/mL. Among the myokines analyzed, there was a significant linear relationship between CPK and myostatin (*Y* = 0.0002917 × X + 1.296, *p* = 0.0014, *r* ^2^ = 0.66). No correlation was found between VO_2_peak, HHb/isch peak and CPK values (*p* = 0.44, *r* ^2^ = 0.07 and *p* = 0.45, *r* ^2^ = 0.08, respectively).

The CTRL group's cardiopulmonary fitness level was considered acceptable if it fell within the “fit” or “sedentary” ranges: 63% were those who had been performing aerobic exercise three to five times/week, 30–60 min each session, for at least 1 year and not involved in regional or national competitions; and 37% were those who had been performing various recreational activities 1–2 times/week, 30–60 min each session, for at least 1 year.

### 3.2 The patterns of breathing

All GSDIII‐p underwent CPET without experiencing any adverse exercise‐related events on the day of evaluation as well as on subsequent days. All GSDIII‐p had to interrupt the CPET due to the inability to maintain the pedaling frequency, despite vigorous encouragement from the researchers. One GSDIII‐p stopped abruptly for a reported respiratory fatigue while the others did so for lower limb fatigue.

Table 3 presents the cut off peak values for each group related to Vt, the InEW and the correlation model of the relationship between V̇E versus Vt (i.e., the presence or not of a non‐linear regression).

CTRL had statically higher values of V̇O_2_ (1.7 ± 0.7 vs. 3.2 ± 1.1 L/min, *p* < 0.0035), V̇CO_2_ (1.6 ± 0.7 vs. 3.5 ± 1.2 L/min, *p* < 0.0004), V̇E (50.5 ± 19.8 vs. 113.6 ± 40.4 L/min, *p* < 0.0005), Vt (1.5 ± 0.5 vs. 2.7 ± 0.8 L, *p* < 0.0013), Rf (32.4 ± 9.1 vs. 42.3 ± 8.1 br/min, *p* < 0.0192), blood lactate (1.8 ± 0.7 vs. 7.6 ± 3.0 mmol/L, p < 0.0004), HR predicted (88.1% ± 2.1% vs. 98.7% ± 4.2%, *p* < 0.0209), HR vs. VO_2_ slope (4.8 ± 1.7 vs. 2.6 ± 0.5, *p* < 0.0007), HHb/isch (39.1% ± 9.9% vs. 74.8% ± 36.6%, *p* < 0.0128). No differences were seen for PETCO_2_ values and BORG scores. (Table 4).

**TABLE 4:** Individual peak data of people with GSD‐III in the four study groups divided according to V̇E values at peak. CTRL group values are expressed as the mean and standard deviation.

	CPET peak values														
	Workload	V̇O_2_	V̇O_2_	V̇O_2_ of pred.	V̇CO_2_	V̇E	Vt	Rf	PETCO_2_	Lactate	BORG	HR of pred.	HR vs. V̇O_2_	V̇E vs. V̇CO_2_	HHb/isch
						BTPS	BTPS								
	W	mL kg^−1^*min^−1^	L min^−1^	%	L min^−1^	L min^−1^	L	br min^−1^	mmHg	mmol L^−1^	Score	%	Slope	Slope	%
Very good responders															
GSDIII9	210.0	36.3	3.0	79	3.0	91.0	2.6	34.5	39.6	3.3	19	88	3.3	25.6	42
Good responders															
GSDIII8	170.0	30.2	3.0	68	2.9	90.0	1.8	40.0	39.3	2.4	16	96	3.1	33.8	60
Poor responders															
GSDIII7	60.0	19.8	1.3	58	1.2	36.9	1.4	21.8	37.0	1.8	19	92	5.2	32.8	45
GSDIII6	114.0	19.2	2.0	47	1.8	59.5	1.6	30.0	34.9	1.7	18	63	6.1	25.8	33
GSDIII5	60.0	21.8	1.6	51	1.4	60.1	1.4	37.8	28.7	1.5	19	73	2.8	26.8	39
Exercise intolerants															
GSDIII4	50.0	13.9	1.1	30	1.1	39.5	0.8	47.0	34.9	1.5	19	87	7.8	31.0	33
GSDIII3	35.0	17.6	1.2	72	1.0	34.5	1.1	31.5	34.2	1.5	17	100	6.8	24.2	38
GSDIII2	45.0	14.4	1.2	35	0.9	32.9	0.8	38.5	32.1	0.7	19	100	4.0	25.3	24
GSDIII1	80.0	18.8	1.6	56	1.4	33.0	1.8	19.2	48.1	1.7	12	94	4.3	25.0	38
CTRL	230.9 ± 90.2*	45.3 ± 12.6*	3.2 ± 1.1*	120.0 ± 15.3*	3.5 ± 1.2*	113.6 ± 40.4*	2.7 ± 0.8*	42.3 ± 8.1*	37.7 ± 3.2	7.6 ± 3.0*	16.2 ± 1.3	98.7 ± 4.2*	2.6 ± 0.5*	26.2 ± 2.5	74.8 ± 36.6*

*Note*: HR vs. V̇O_2_ and V̇E vs. V̇CO_2_ are slopes of the relationship between the respective outcomes; HHb/isch = peak value of NIRS measurments (see text for further explanations). *p < 0.05 when compared to the averaged values of GSDIII‐p.

Abbreviations: BORG, rating of perceived exertion; HR, heart rate; PETCO_2_, pulmonary end tidal breath CO_2_; Rf, respiratory frequency; V̇CO_2_, carbon dioxide production; V̇E, pulmonary ventilation; V̇O_2_, oxygen uptake; Vt, tidal volume.

**FIGURE 1:**
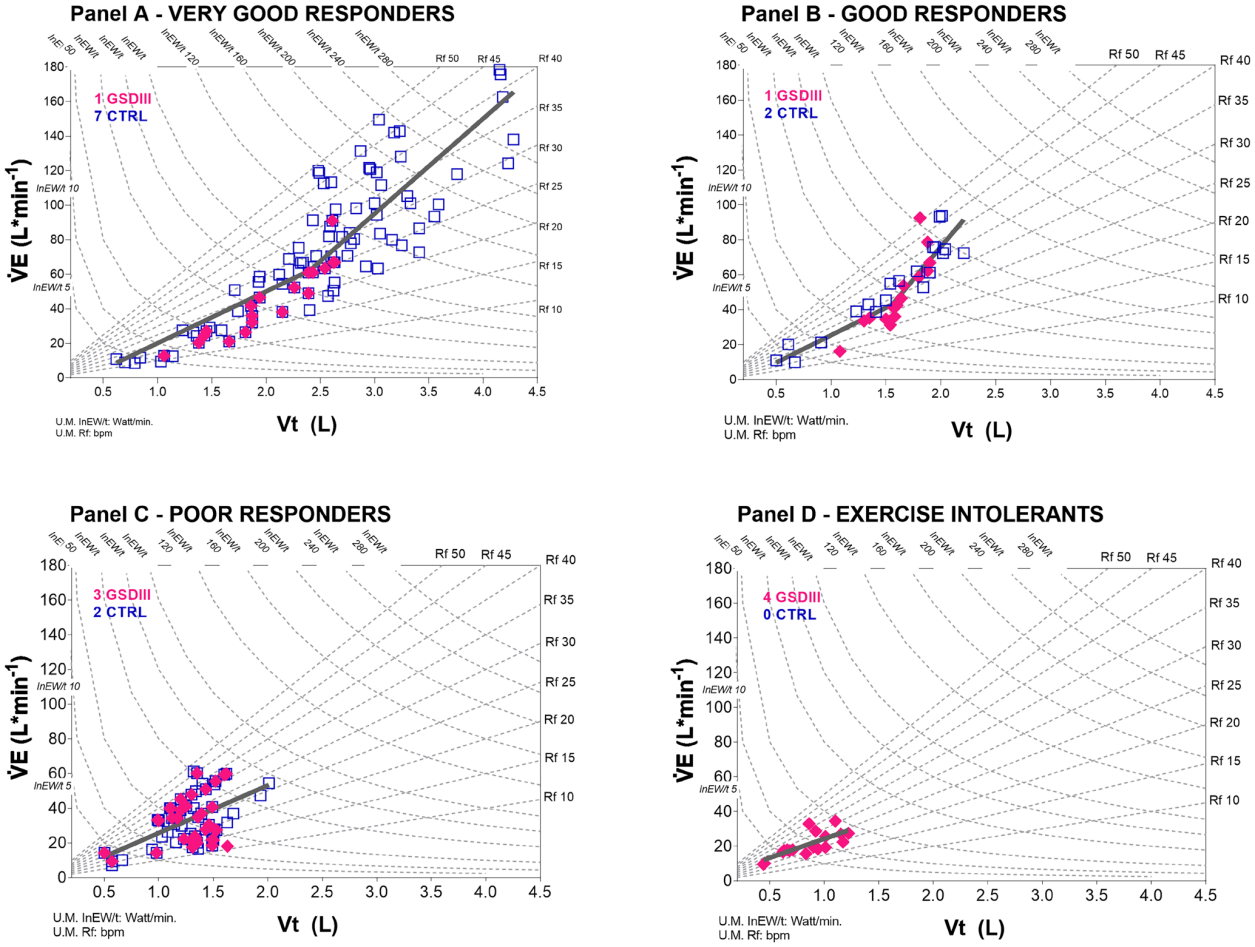
The relationship between pulmonary ventilation (V̇E) and tidal volume (Vt) is shown during cardiopulmonary exhaustion testing. The iso‐respiratory frequency lines (Rf) range from 10 to 50 breaths per minute (bpm). The iso‐inspiratory power lines (InEW/t) range from 5 to 280 watts per minute (watt/min). Individual values are shown for GSDIII‐p (full rhombus) and CTRL (empty squares). Panels A through D show the individual strategies to cope with exercise from resting condition to peak workloads. The four study groups, which are divided according to V̇E values at peak, exhibit different patterns of breathing: Very good responders (Panel A), good responders (Panel B), poor responders (Panel C), and exercise intolerants (Panel D). Averaged nonlinear regressions of V̇E versus Vt are shown in panels A and B (continuous lines), and averaged linear regressions of the same relationship are shown in panels C and D.

Figure 1 shows the relationship between V̇E and Vt in GSDIII‐p and CTRL during incremental exercise. Figure 1 also shows the iso‐respiratory frequency (Rf) and iso‐inspiratory elastic power lines (InEW/t). In panel A, the strategies of the very good responders (7 CTRL and 1 GSDIII‐p) to sustain the incremental exercise are presented: the increase in V̇E to support the increase in V̇O_2_, according to the increasing workload, was achieved through an initial increase in both Vt and Rf and a final remarkable increase in just Rf to maintain the highest V̇E. When the data from these eight participants were grouped, the best fit for the V̇E vs. Vt relationship was a two‐linear regression with a Vt threshold at 2.4 L corresponding at 82% of the averaged Vt peak. The peak InEW/t range was between 120 and above 290 W. The good responders to exercise (panel B): 1 out of 3 was a GSDIII‐p that exhibited similar strategies to those of very good responders: a non‐linear regression with a Vt threshold was calculated on average for the few participants in this pattern of breathing (Vt 1.5 L at 78% of Vt peak). The peak InEW/t range was between 80 and above 120 W.

The majority of GSDIII‐p fell into panels C (poor responders) and D (exercise intolerant). A significant reduction in V̇E peak was shown in these groups, likely due to the higher Rf from the start of exercise and possibly due to poor Vt values, resulting in a low peak InEW/t (range 15–50 W, panel D and 60–80 W panel C). Two CTRL also fell in panel C. For these patterns of breathing, the Vt threshold was not identified when all 9 participants' data were grouped: a linear regression of the relationship between V̇E vs. Vt was the preferred model (see Table 2).

### 3.3 The alveolar ventilation

**FIGURE 2:**
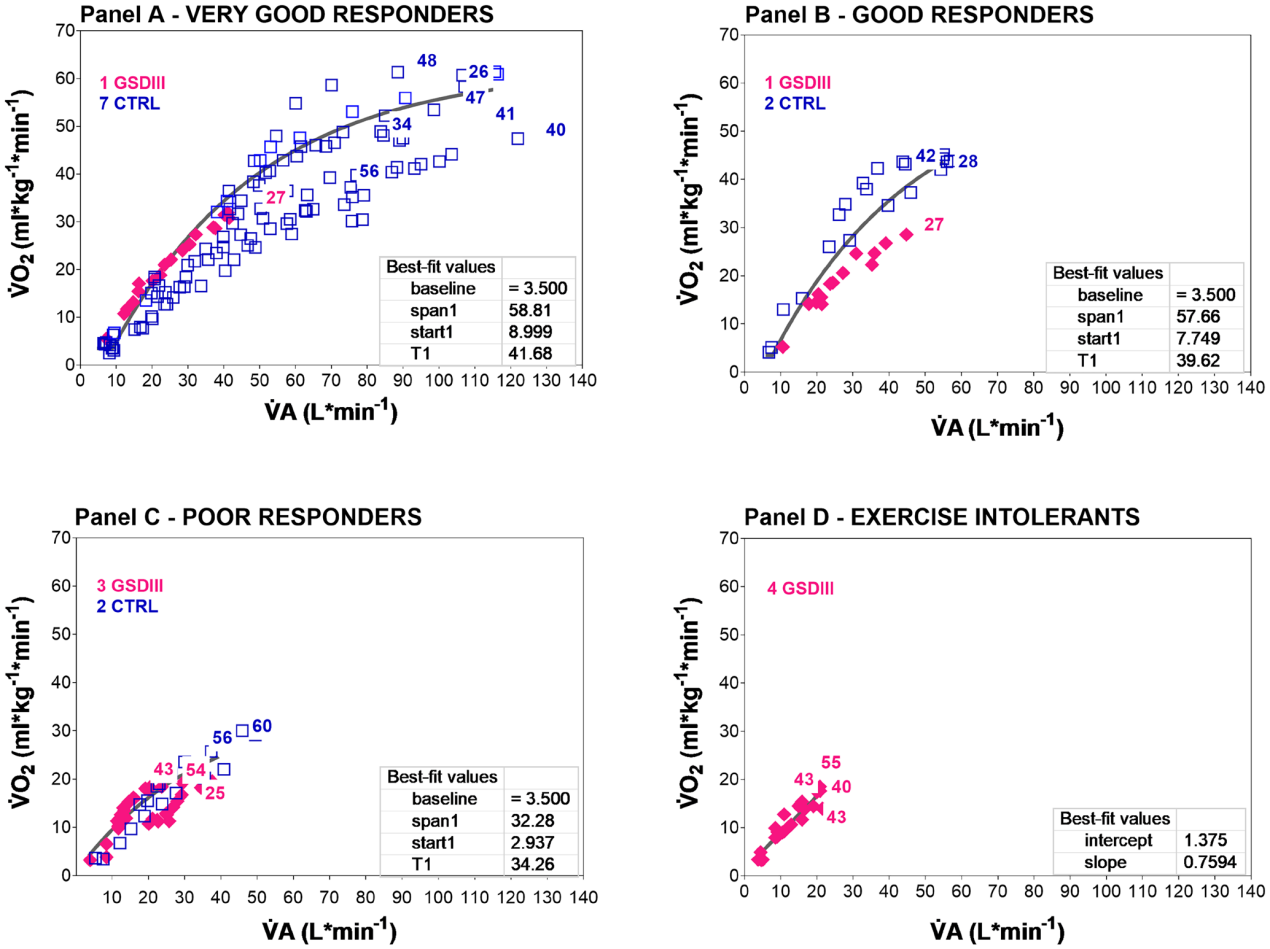
The relationship between oxygen uptake (V̇O_2_) and alveolar ventilation (V̇A) is shown during cardiopulmonary exhaustion testing. Individual values are shown for GSDIII‐p (full rhombus) and CTRL (empty squares). Panels A through D show the individual strategies to cope with exercise from resting condition to peak workloads. The four study groups, which are divided according to V̇E values at peak, exhibit different patterns of breathing: very good responders (Panel A), good responders (Panel B), poor responders (Panel C), and exercise intolerants (Panel D). The age of each participant is placed over the corresponding individual peak value. Averaged mono‐exponentials are shown as the best fit for the V̇O_2_ vs. V̇A relationships in panels A–C. Panel D shows a linear regression as the best fit. All relationships are presented as continuous lines.

Figure 2 shows the relationship between V̇O_2_ and V̇A in GSDIII‐p and CTRL during incremental exercise. The same 4 patterns of breathing were used to identify how the V̇A adapted to exhaustive exercise. Among the individual patterns of breathing of each panel the best fit of the V̇O_2_ vs. V̇A relationship was a mono‐exponential line, with an exception for panel D (linear regression). Lower spans of V̇O_2_ (between resting and peak) and shorter time of exercise are characteristic of poor responders and exercise intolerants (panels C and D) when compared to very good and good responders (panels A and B). For GSDIII‐p, older age was not the limit in reaching a certain percentage of normalized V̇O_2_ peak for age, gender, and body mass; the youngest GSDIII‐p did not reach V̇O_2_ peak values higher than the oldest CTRL and GSDIII‐p.

### 3.4 PETCO_2_ and exercise tolerance

**FIGURE 3:**
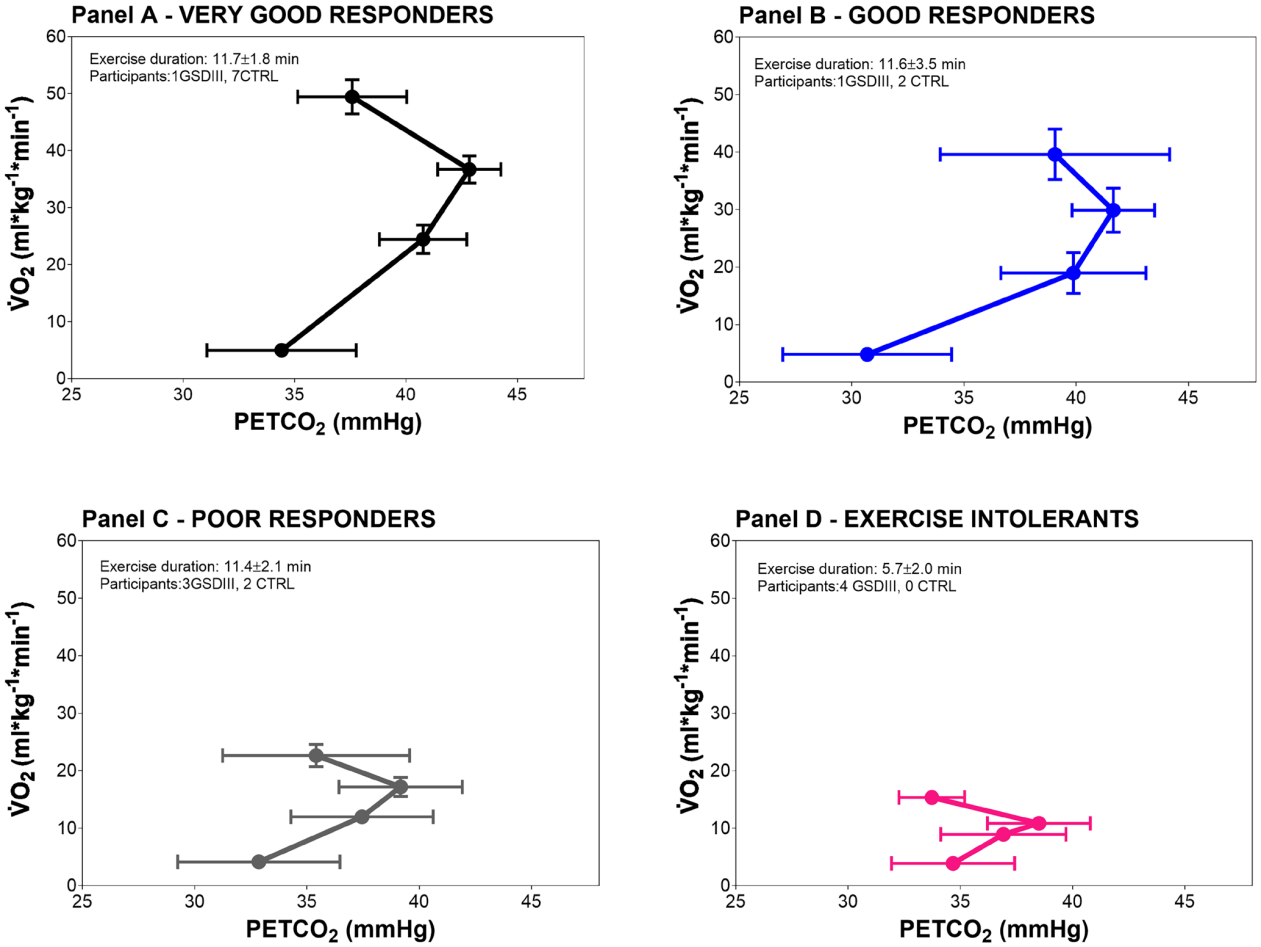
Connecting lines are shown during cardiopulmonary exhaustion testing that connect the averaged values of oxygen uptake (V̇O_2_) and end‐tidal CO_2_ (PetCO_2_) at rest, at 50%, at 75% and at the peak of exercise capacity. The averaged values and standard error of the mean (SEM) are shown. Panels A through D depict strategies for coping with exercise, from rest to peak workloads. The four study groups, which are divided according to V̇E values at peak, exhibit different patterns of breathing: Very good responders (Panel A), good responders (Panel B), poor responders (Panel C), and exercise intolerants (Panel D).

Figure 3 shows the changes in V̇O_2_ and PETCO_2_ at rest and at −50%, −75%, and −100% of peak exercise. The same 4 patterns of breathing were used to identify how PETCO_2_ adapted to exhaustive exercise. Values are averaged for all CTRL and GSDIII‐p at each point in each panel. In all panels, the increase in V̇O_2_ with increasing workload was achieved through an initial increase in PETCO_2_, followed by a remarkable decrease in PETCO_2_ from 75% of peak exercise to exhaustion. On average, participants in Panels C and D did not exceed a PETCO_2_ cutoff point of 40 mmHg during all CPET. On average the final values of PETCO_2_ were not statistically different from those of CTRL (Table 4).

### 3.5 The skeletal muscle fractional O_2_ extraction

**FIGURE 4:**
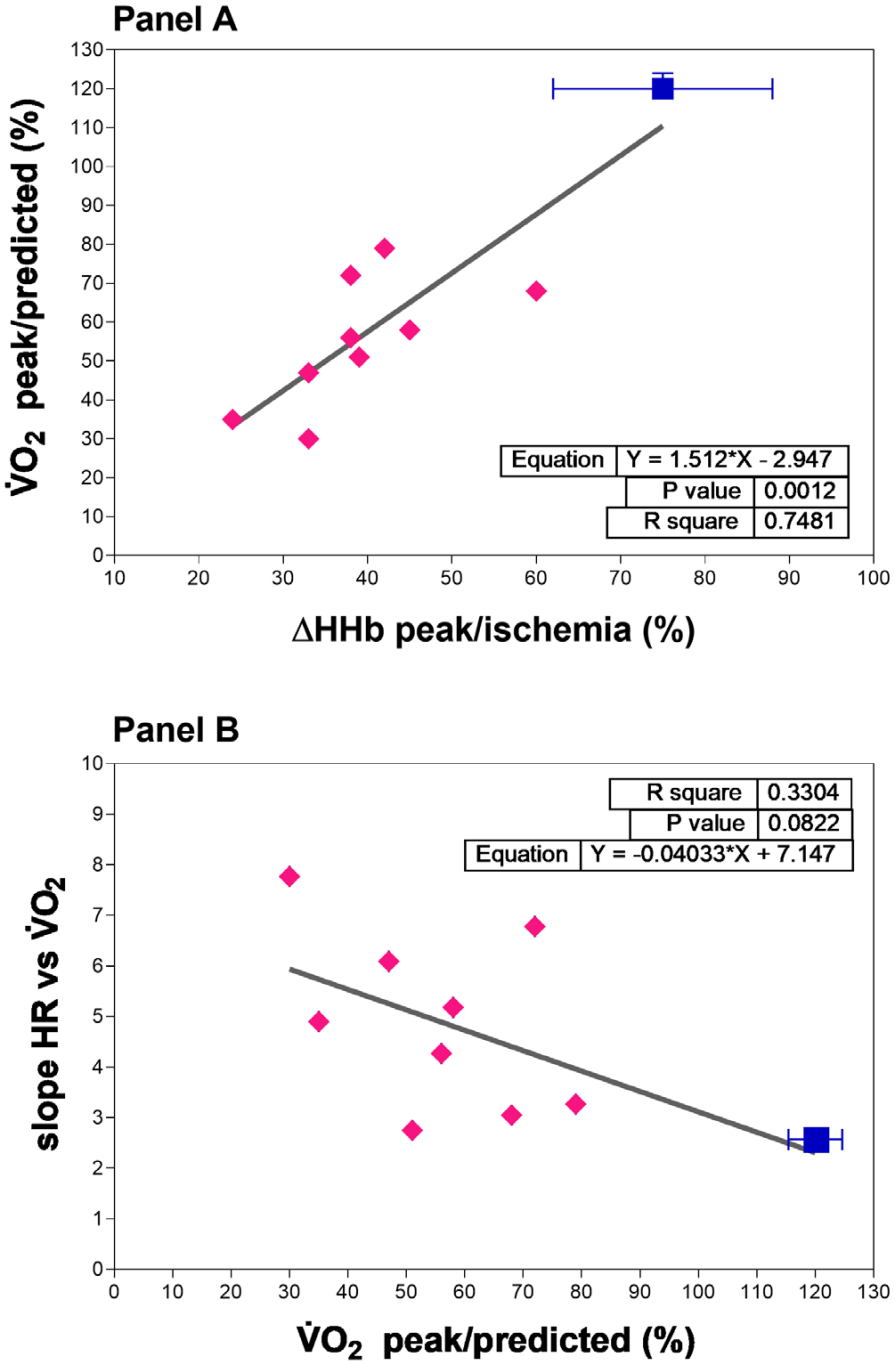
Panel A shows the linear regression between peak oxygen uptake (V̇O_2_peak) and skeletal muscle oxygen extraction (HHbpeak)/ischemia), as measured by near‐infrared spectroscopy. Panel B shows the linear regression between the cardiovascular response to workload (slope of HR/V̇O_2_) and V̇O_2_peak. Individual values are shown for people with GSD‐III (full magenta rhombus), while the CTRL group is represented by the average of both variables and their variances (full blue square). The averaged best fit of the relationships is shown as a continuous line in both panels.

Figure 4, panel A shows a significant linear relationship between V̇O_2_peak expressed as percentage of predicted values (Wasserman‐Hansen equation) for age, sex and body weight (V̇O_2_peak/predicted) and HHbpeak compared to maximal ischemic values (HHbpeak/isch) in GSDIII‐p: higher HHbpeak/isch corresponds to higher V̇O_2_peak/predicted values (*p* = 0.0012, *r* ^2^ = 0.748, *Y* = 1.512*x* − 2.947). The CTRL group is represented as averaged values for both variables, along with their variances.

There was a tendency towards a significant linear relationship (Figure 4, panel B) between the individual slopes of the HR vs. V̇O_2_ relationships and V̇O_2_peak/predicted in GSDIII‐p: higher HR/V̇O_2_ slopes correspond to higher V̇O_2_peak/predicted values (*p* = 0.0822, *r* ^2^ = 0.330, *Y* = −0.040*x* + 7.147). CTRL group is represented as averaged values for both variables with their variances.

### 3.6 Functional evaluation

**FIGURE 5:**
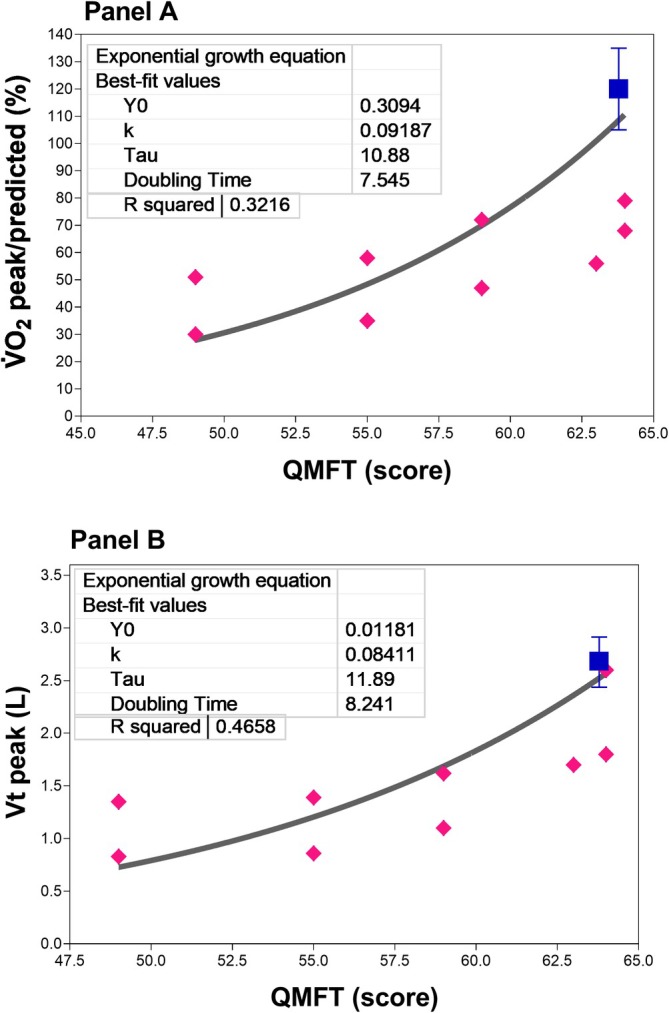
Panels A and B show the exponential growth relationships between peak oxygen uptake (V̇O_2_ peak) and tidal volume (Vt) and quick motor function test (QMFT) scores. Individual values are shown for people with GSD‐III (full magenta rhombus), while the CTRL group is represented by the average of both variables and their variances (full blue square). The averaged best fit of the relationships is shown as a continuous line in both panels.

Figure 5 shows the non‐linear relationship between V̇O_2_ peak/predicted (Panel A) and Vt peak (Panel B) versus QMFT scores. The CTRL group is represented by the average of both variables and their associated variances. The exponential curve in panel A shows that higher V̇O_2_peak/predicted values corresponds to higher QMFT scores. The same applies to panel B where higher Vtpeak values correspond to higher QMFT scores. For the same Vt or V̇O_2_peak/predicted values, there are QMFT scores ranging from normal to impaired functional capacity in both panels.

## 4 DISCUSSION

The novelty of this study lies in its perspective on describing the GSDIII‐p response to exercise, thereby obtaining an overall physiological picture of the complex interplay among the respiratory, cardiovascular, and skeletal muscle systems. When the body's increased energy demand challenges the efficiency of the O_2_ transport and utilization chain, GSDIII‐p try to meet the increased metabolic demand by exploiting compensatory strategies from different systems. GSDIII‐p suffer from the consequences of their disease (Kishnani et al., 2010) that impact several systems and therefore lead to a wide spectrum of functional impairment ranging from response to exercise similar to healthy individuals to exercise intolerance. Our results show that: (1) there are 4 different patterns of breathing ranging from exercise intolerance to very good response; (2) alveolar ventilation is reduced in exercise intolerance; (3) skeletal muscle extraction by NIRS is limited in those with reduced exercise capacity.

Some of the observed responses may not be exclusively specific to GSDIII‐p, but rather represent a common pattern of exercise intolerance associated with various diseases, such as metabolic myopathies (Grassi et al., 2007) and motor neuron diseases (Lanfranconi et al., 2017), as well as in the older adults (Wyckelsma et al., 2017). These responses may also be influenced by reduced fitness levels or muscle weakness related to other conditions, such as bed rest (Porcelli et al., 2010).

Because similar considerations are applied to other diseases, for which the usefulness of widely used, non‐invasive approaches to identify and quantify impaired skeletal muscle oxidative metabolism, the factors limiting exercise tolerance, and, ultimately, patient quality of life (QoL) have been highlighted by several authors (Preisler, Haller & Vissing, 2015; Ferri et al., 2019; Grassi et al., 2020; Lanfranconi et al., 2020), we thought that, in order to be in line with this approach, patients with GSDIII‐p were evaluated using a comparable framework.

### 4.1 Anthropometric findings and clinical characteristics

GSDIII‐p presented a higher percentage of fat mass when compared to CTRL. This finding may be related to a degenerative mechanism that results in an atrophic muscle with increased fat mass exacerbated by the sedentary behavior (Wary et al., 2010). In their study, Wary and colleagues (Wary et al., 2010) found an excess accumulation of glycogen, and nuclear magnetic resonance imaging revealed progressive fat infiltration that paralleled the muscle weakness.

A consensus guideline published in 2010 suggested using corn starch to prevent hypoglycemia, a high protein intake in infants and children with GSDIII, and a regular, well‐balanced diet in adults (Kishnani et al., 2010). Even though GSDIII‐p involved in this study followed a proper diet from their infancy, one developed liver fibrosis and cirrhosis, and all (with only one exception) showed signs of possible chronic muscle damage (i.e., increased blood CPK), probably due to progressive storage of abnormal glycogen (Kishnani et al., 2010; Berling et al., 2021). In this study, possible skeletal muscle damage was paralleled by elevated myostatin values. Myostatin naturally increases with age, sarcopenia, cachexia, and bed rest, leading to decreased muscle mass and strength (Buehring & Binkley, 2013). No previous data relating CPK and myostatine levels in GSDIII‐p are available. The ratios of log10 myostatin‐to‐CPK, as well as albumin‐to‐myostatin, are biomarkers utilized to identify patients with sarcopenia with fairly good diagnostic accuracy (Alexopoulos et al., 2021). No meaningful relationship was found when considering the other three proposed cytokines/myokines, perhaps also due to the confounding fact that they are produced by different sources beside skeletal muscle (e.g., immune system cells for IL‐6 and TNF‐α). This may be also the case of BDNF that is stocked in platelets and released upon aggregation, albeit a previous study found a relationship between disease stage and BDNF serum levels in amyotrophic lateral sclerosis, a neurodegenerative condition characterized by muscle wasting (Tremolizzo et al., 2016). Indeed, there is evidence that a high‐fat diet reduces plasma CPK concentrations and has a beneficial effect on cardiac hypertrophy in children (Rossi et al., 2020).

As possible considerations on cardiac output efficiency, in this study the LVMI was slightly increased, although the ejection fraction remained normal in all GSDIII‐p. Although echocardiography was not performed during CPET, a hemodynamically significant valvopathy and/or a dynamic outflow obstruction during exercise can be reasonably excluded. None of the GSDIII‐p showed arrhythmias at rest or during exercise. Therefore, in our GSDIII‐p, we could reasonably assume that the cardiac output was not a limitation to their cardiopulmonary performance.

### 4.2 The heterogenic patterns of breathing in GSDIII‐p

This study was inspired by a previous analysis by the same research group on patterns of breathing in people with amyotrophic lateral sclerosis (Lanfranconi et al., 2017). There is a lack of knowledge about the respiratory strategies that GSDIII‐p could utilize in different stages of the disease to sustain strenuous exercise. The energetics of respiratory muscles in GSDIII‐p have always been somehow overlooked. There is a general misconception that respiratory muscles behave like other skeletal muscles; however, even if some general principles can be derived, the peculiar requirements of the respiratory muscles caution against the assumption that they behave in the same way as all other skeletal muscles (Roussos & Koutsoukou, 2003; Aboussouan, 2009; Aliverti, 2016). Respiratory failure in neuromuscular disorders is thought to result from both progressive weaknesses of the inspiratory muscles and increased work of breathing due to increased stiffness of the lungs and chest wall (Lechtzin et al., 2006). Tobaly et al. (2019) described the radiological pattern of muscle involvement in a cohort of GSDIII‐p. Their findings suggest that most patients with GSDIII have a characteristic pattern of muscle involvement on MRI including respiratory muscles with a clear narrowing of the pulmonary apex, probably due to fatty impairment and infiltration of the diaphragm with sparing of the intercostal muscles. To our understanding in our cohort of GSDIII‐p, under these conditions, the respiratory system remains capable of adapting and triggering compensatory respiratory plasticity mechanisms, at least in the early stages, which preserve breathing capacity (i.e., the balance between metabolic CO_2_ production and disposal) despite the loss of respiratory muscle function.

When exercise becomes strenuous, it may force GSDIII‐p, especially those defined as poor responders and exercise intolerants, to adopt a less energy demanding strategy in terms of patterns of breathing, relying more on the increase of Rf than on the increase of Vt. In healthy individuals, the respiratory muscles (particularly the diaphragm) mainly rely on the oxidative energy production pathway. Whenever the respiratory muscles must perform very high respiratory mechanical power, they must inevitably exploit more phosphorylation at substrate level for ATP production to assure adequate ventilation before becoming exhausted. This chain of events, though, does not occur in GSDIII‐p because the glucose they manage to transport into circulation is much less than the amount provided by healthy individuals. They attempt to compensate for the energy deficit by using alternative substrates such as lactate and ketone bodies. However, in this study, GSDIII‐P had limited muscular lactate production, probably due to a shortage of substrates for anaerobic glycolysis. This resulted in imbalanced blood lactate production and utilization, ultimately leading to poor mechanical ventilation.

There is an optimal respiratory frequency for a given level of V̇E at which the least amount of energy is expended for a given V̇E. In CTRL, the choice of increasing V̇E by relying primarily on Rf only occurs when approaching the highest ventilatory rate. The InEW/t is inversely related to Rf, whereas non‐elastic work is independent of Rf. It then follows that, for a given V̇E, the work of breathing should be lower the higher Rf is. Indeed, for moderate levels of V̇E (5–35 L/min) and for a range of 5–20 breaths/min, the O_2_ cost of breathing diminishes as the Rf increases. The decrease in work of breathing, which occurs in parallel with the rise in Rf, has, however, a limit. With a high V̇E, requiring active participation of the expiratory muscles, the total work, which becomes mainly non‐elastic, becomes independent of Rf. Furthermore, by increasing the Rf, the mechanical efficiency may appreciably decrease, thus increasing V̇O_2_. Therefore, it appears that GSDIII‐p, characterized by less efficient patterns (very low Vt peaks and high Rf), demonstrates an uneconomical strategy that increases V̇O_2_, leading to early exhaustion.

As for the inefficient pattern of breathing for two CTRL, their sedentary behavior could likely explain it, but a possible unknown pre‐clinical disease might also be suspected.

### 4.3 Alveolar ventilation and exercise at exhaustion

When evaluating the efficiency of the respiratory system, one can focus on V̇A rather than V̇E because Vt is far more effective than an increase in respiratory rate (i.e., Rf) in elevating V̇A. During exercise in healthy mammals, V̇A and alveolar‐capillary diffusion capacity increase in proportion to the increase in metabolic rate to prevent an increase in PaCO_2_ and a decrease in PaO_2_ (Forster et al., 2012). To a certain extent, exercise can exacerbate the mechanisms that lead to failure of the gas exchanges usually due to ventilatory failure, manifested by hypercapnia and/or hypoxaemia (Roussos & Koutsoukou, 2003). Individual patterns of breathing during exercise, that is, the successful or unsuccessful strategies used to cope with V̇A for a given V̇CO_2_ (as a result of increased O_2_ demand from oxidative metabolism), can be used, combined with the clinical history and assessment, as a general guide to understand the severity of the disease.

Exercise is a strong edemogen condition as the increase in cardiac output leads to lung capillary recruitment for fluid exchange and potential increase in capillary pressure (Miserocchi & Beretta, 2023). The physiological low microvascular permeability may be impaired by intensity and duration of exercise, causing damage to the interstitial matrix macromolecular assembly leading to alveolar edema and hemorrhages accordingly to inter‐individual proneness to develop lung edema (Miserocchi & Beretta, 2023). In CTRL, the modulation of V̇A in relation to V̇O_2_ is essential to maintain stable partial pressures of gases in the alveoli, and therefore the pressure gradients that sustain gas exchange. A loss of linearity in this relationship (Figure 2) suggests an overproduction of V̇CO_2_, which is not of metabolic origin, but results from lactate buffering (with an increase in the respiratory equivalent for V̇O_2_ and a constant respiratory equivalent for V̇CO_2_) or pH compensation.

On the other hand, in GSDIII‐p, V̇A was lower with respect to the CTRL group, which could suggest a limitation in O_2_ consumption by the respiratory muscles or an increase in resistive respiratory work. Apparently, the low V̇A values of GSDIII‐p with less efficient patterns of breathing are suggestive of a predisposition towards an edemogen agent of uncertain cause that leads to lower exercise tolerance. GSDIII‐p presented an earlier increase in Rf with respect to the CTRL in the attempt to reduce InEW as early as possible. This strategy may be used to avoid exceeding a certain Vt to prevent crossing the alveolar folding/unfolding zone, which could reduce lung compliance and increase further the elastic work (Miserocchi & Beretta, 2023).

### 4.4 End tidal pressure of CO_2_ and exercise performance

PETCO_2_ changes have been described in individuals with heart failure and acute pulmonary embolism, as well as in pulmonary hypertension. During exercise, PETCO_2_ may be considered as a non‐invasive indicator of arterial CO_2_ and a marker of V̇E efficiency and cardiac function. In healthy individuals, PETCO_2_ generally increases or remains stable at the start of an incremental exercise, then declines at higher workloads following the increased V̇E. In individuals with heart failure, however, PETCO_2_ may decrease significantly due to impaired cardiac output, poor muscle perfusion, and an increased ventilation‐perfusion mismatch.

A similar behavior was seen in GSDIII‐p, with decreasing values at peak workloads in all breathing patterns. Additionally, the time spent between 75% and 100% exercise intensities was dramatically shorter in participants shown in panels C and D (Figure 3), although the final attempt to compensate for increasing PETCO_2_ was the same.

The role of blood lactate in increasing PETCO_2_ is complex, particularly in individuals who do not respond well to exercise. However, during exercise, lactate production, utilization, and elimination are usually balanced (Stanley, 1991). GSDIII‐p are typically considered non‐lactate producers, and all 3 processes may be impaired, even though some GSDIII‐p presented lactate production, a finding indicating that they may partially exploit cytosolic substrate phosphorylation for ATP production and provide tissues and organs with lactate as a substrate to produce energy aerobically. The most exercise‐intolerants, however, did not exhibit higher circulating lactate levels even when they were exhausted, indicating that they were probably unable to produce it. Furthermore, the cardiac pump's use of lactate and respiratory compensatory mechanisms may prevent the accumulation of undetected lactate at the end of exercise.

### 4.5 The skeletal muscle O_2_ extraction

Our findings follow the path suggested by Preisler et al. (2013) according to whom the general classification of GSD III as a glycogenosis characterized by fixed symptoms related to muscle wasting should be modified to consider dynamic exercise‐related symptoms of muscle fatigue including insufficient energy production in muscle (Preisler et al., 2013). The impaired capacity of O_2_ extraction by skeletal muscle in GSDIII‐p, as evaluated by NIRS, can therefore yield pathophysiological insights, unveiling peripheral metabolic impairment contributing to the reduced exercise tolerance. In addition, it can offer diagnostic clues as already shown in metabolic myopathies (Grassi et al., 2007, 2019).

The pathophysiology underlying muscle dysfunction in GSDIII‐p is still incompletely understood (Hoogeveen et al., 2021). In young adults with GSDIIIa, the myopathy usually presents as exercise intolerance with both proximal and distal muscle involvement and elevated blood markers of myolysis. Muscle weakness/wasting and contractile mass loss (dysfunctional autophagy) are reported as a recurrent symptom in these individuals, demonstrating that myopathy may occur earlier than usually reported (Kishnani et al., 2010; Laforêt et al., 2019; Wary et al., 2010). In addition, as a likely consequence of exercise intolerance, many GSDIII‐p lead a sedentary lifestyle, which itself is associated with unwanted metabolic adaptations and further health issues (Bordoli et al., 2022). Last, in vivo findings of delayed intramuscular metabolic recovery post exercise suggest that an inefficient oxidative metabolism may also result from cellular energy crisis during exercise as a result of reduced mitochondrial capacity for oxidative ATP synthesis (Wary et al., 2010).

Accordingly to Tobaly et al. (2019) GSDIII‐p have the second most frequent cause of glycogenosis with fixed muscle weakness after Pompe disease: in their study the authors showed a peculiar pattern of muscle involvement with whole‐body MRI, especially distal leg muscle involvement. Imaging that can be correlated with clinical and functional parameters, such as those found in our study, may contribute to the development of a severity score that improves GSDIII‐p management. Furthermore, regarding microcirculation/endothelial function and mitochondrial impairment, this study lacks the experimental data required to qualitatively distinguish which factor may impact the oxidative metabolism more. Poole et al. found that a plethora of structural and functional impairments (in terms of neurohumoral, inflammatory and reflex processes) in muscle microcirculations could explain the reduced skeletal muscle oxidative function in persons with chronic heart failure (Poole et al., 2012). We suspect that something similar could be true for GSDIII‐p: further studies on mitochondrial respiration in this population will help to clarify the issue.

The QMFT is a functional motor scale specifically designed for individuals with Pompe disease, which is easy to apply and sufficiently sensitive to detect clinically relevant changes in the follow up evaluation (van Capelle et al., 2012). We thought that the QMFT was easy to use in GSDIII‐p. A fair relationship between V̇O_2_ values and QMFT scores was observed, but especially the participants most intolerant to exercise had different characteristics not truly evident by considering just the QMFT scores. In GSDIII‐p, there is a need to identify additional treatments to complement the diet and further improve their health outcomes. One such intervention could be exercise training programs tailored to the specific pathophysiology of this population. A functional evaluation as part of their follow‐up will help improve a new clinical approach to glycogenosis.

## 5 CONCLUSION

People with GSDIII display features of a “human knockout” model, providing unique opportunities to investigate fundamental physiological links between systems involved in individual exercise tolerance. Our study shows that in GSDIII‐p the impairment of oxidative metabolism, especially the respiratory and skeletal muscle systems, can ultimately limit exercise capacity. The non‐invasiveness of the adopted methods can facilitate serial measurements, allowing the clinical path of the diseases to be examined, as well as the efficacy of pre‐rehabilitation or nutritional end exercise–based interventions.

The physiological responses of these GSDIII‐p during exercise proved to be quite heterogeneous, whereas a more homogeneous profile is usually observed in resting conditions. This further supports the validity of adapted exercise as a valuable tool for obtaining a more precise characterization of individuals who appear similar. Conceivably, this is a pivotal information for those clinicians aiming at implementing personalized medicine programs. This study could pave the way for generating high‐quality knowledge about the impact of possible pharmacological or non‐pharmacological interventions (e.g., diet and/or exercise) aimed at maintaining or increasing the exercise capacity of people with GSD‐III.

## AUTHOR CONTRIBUTIONS

The contributions of each author to the conception and design of the research were as follows: F. Lanfranconi, L. Peli, L. Pollastri, A. Ferri, E. Conti, L. Tremolizzo, F. Pieruzzi, G. Miserocchi, E. Beretta, M. Marzorati, W. Zardo, S. Gasperini, R. Pretese, S. Paci, C. Capelli, R. Mariani, A. Cattoni, A.C. Balduzzi, R. Parini. Analyzed data (F. Lanfranconi, L. Peli, L. Pollastri, E. Conti, L. Tremolizzo, F. Pieruzzi, M. Marzorati, W. Zardo, R. Pretese). Performed experiments (F. Lanfranconi, L. Pollastri, E. Conti, L. Tremolizzo, F. Pieruzzi, M. Marzorati, W. Zardo, R. Pretese). Interpreted results of experiments (F. Lanfranconi, L. Pollastri, A. Ferri, E. Conti, L. Tremolizzo, F. Pieruzzi, G. Miserocchi, E. Beretta, M. Marzorati, W. Zardo, S. Gasperini, R. Pretese, S. Paci, C. Capelli, R. Mariani, A. Cattoni, A.C. Balduzzi, R. Pretese). Prepared figures (F. Lanfranconi, L. Peli, A. Ferri, G. Miserocchi, E. Beretta, M. Marzorati, W. Zardo, R. Pretese). Drafted manuscript (F. Lanfranconi, L. Pollastri, A. Ferri, E. Conti, L. Tremolizzo, F. Pieruzzi, G. Miserocchi, E. Beretta, M. Marzorati, W. Zardo, S. Gasperini, R. Pretese, S. Paci, C. Capelli, R. Mariani, A. Cattoni, A.C. Balduzzi, R. Pretese). Edited and revised manuscript (F. Lanfranconi, L. Peli, L. Pollastri, A. Ferri, E. Conti, L. Tremolizzo, F. Pieruzzi, G. Miserocchi, E. Beretta, M. Marzorati, W. Zardo, S. Gasperini, R. Pretese, S. Paci, C. Capelli, R. Mariani, A. Cattoni, A.C. Balduzzi, R. Pretese). Approved final version (F. Lanfranconi, L. Pollastri, A. Ferri, E. Conti, L. Tremolizzo, F. Pieruzzi, G. Miserocchi, E. Beretta, M. Marzorati, W. Zardo, S. Gasperini, R. Pretese, S. Paci, C. Capelli, R. Mariani, A. Cattoni, A. Balduzzi, R. Pretese).

## FUNDING INFORMATION

The work reported in this manuscript was not funded/sponsored.

## CONFLICT OF INTEREST STATEMENT

The authors declare no conflicts of interest.

## ETHICS STATEMENT

The study was approved by the local Ethics Committee, and informed consent was obtained from all participants.

## Data Availability

The data that support the findings of this study are available on request from the corresponding author.
